# Bisphosphonates antagonise bone growth factors' effects on human breast cancer cells survival

**DOI:** 10.1038/sj.bjc.6601009

**Published:** 2003-07-01

**Authors:** O Fromigue, N Kheddoumi, J-J Body

**Affiliations:** 1 Laboratory of Endocrinology, Bone Diseases and Breast Cancer Research, Department of Medicine, Institut Jules Bordet, Université Libre de Bruxelles. Rue Heger-Bordet, 11000 Brussels, Belgium

**Keywords:** bisphosphonates, bone metastases, growth factor, breast cancer, apoptosis

## Abstract

Bone tissue constitutes a fertile ‘soil’ for metastatic tumours, notably breast cancer. High concentrations of growth factors in bone matrix favour cancer cell proliferation and survival, and a vicious cycle settles between bone matrix, osteoclasts and cancer cells. Classically, bisphosphonates interrupt this vicious cycle by inhibiting osteoclast-mediated bone resorption. We and others recently reported that bisphosphonates can also induce human breast cancer cell death *in vitro*, which could contribute to their beneficial clinical effects. We hypothesised that bisphosphonates could inhibit the favourable effects of ‘bone–derived’ growth factors, and indeed found that bisphosphonates reduced or abolished the stimulatory effects of growth factors (IGFs, FGF-2) on MCF-7 and T47D cell proliferation and inhibited their protective effects on apoptotic cell death *in vitro* under serum-free conditions. This could happen through an interaction with growth factors' intracellular phosphorylation transduction pathways, such as ERK1/2-MAPK. In conclusion, we report that bisphosphonates antagonised the stimulatory effects of growth factors on human breast cancer cell survival and reduced their protective effects against apoptotic cell death. Bisphosphonates and growth factors thus appear to be concurrent compounds for tumour cell growth and survival in bone tissue. This could represent a new mechanism of action of bisphosphonates in their protective effects against breast cancer-induced osteolysis.

Breast and prostate cancers most often metastasise to bone (Yoneda *et al*, 2000). A ‘seed and soil’ hypothesis (Paget, 1889) can explain this phenomenon. The bone extracellular matrix is rich in growth factors (Hauschka *et al*, 1986), which are released during the continuous remodelling process and favour cancer cell proliferation and survival (Barnes, 1988; Geier *et al*, 1992; Quinn *et al*, 1996). On the other hand, breast cancer cells are able to stimulate bone resorption by increasing osteoclast recruitment and proliferation as well as the activity of mature osteoclasts (Mundy, 1991; Taube *et al*, 1994; Yoneda *et al*, 2000). Bone microenvironment will then be even more enriched in bone-derived growth factors that enhance proliferation and survival of cancer cells. This sets up a vicious cycle between cancer cells, osteoclasts and bone matrix.

Bone metastases are responsible for considerable misery in cancer patients. Bisphosphonates reduce the skeletal morbidity rate in breast cancer patients with bone metastases by up to 40–50% (Paterson *et al*, 1993; Body *et al*, 1998; Hortobagyi *et al*, 1998; Theriault *et al*, 1999; Body, 2001) and they can decrease the number and the extent of bone metastases in animal models of tumour-induced osteolysis (Hughes *et al*, 1995; Hiraga *et al*, 2001) as well as in patients treated in the adjuvant setting (Coleman, 2002; Powles *et al*, 2002).

Bisphosphonates are synthetic analogues of pyrophosphate in which the oxygen bridge is replaced by a carbon atom that allows the attachment of various side chains. Bisphosphonates are very stable compounds that exhibit a high affinity for calcified matrices such as hydroxyapatite in bone (Lin, 1996), and are successfully used as powerful inhibitors of increased bone resorption in several bone diseases (Fleisch, 1997a, 1997b). They act by decreasing the recruitment, proliferation and differentiation of preosteoclasts (Lowik *et al*, 1988; Hughes *et al*, 1989; Nishikawa *et al*, 1996), their adhesion to the mineralised matrix and, most importantly, the resorptive activity of mature osteoclasts (Sato *et al*, 1991; Selander *et al*, 1994; Azuma *et al*, 1995; Murakami *et al*, 1995). They also shorten osteoclast lifespan by induction of programmed cell death (apoptosis) (Hughes *et al*, 1995). In addition, the inhibitory activity of bisphosphonates on bone resorption may be indirectly mediated by other cells such as cells of the osteoblastic lineage or the macrophage family (Sahni *et al*, 1993; Nishikawa *et al*, 1996; Vitte *et al*, 1996; Siwek *et al*, 1997; Fromigue and Body, 2002). On the other hand, we and others (Fromigue *et al*, 2000; Senaratne *et al*, 2000; Jagdev *et al*, 2001a) previously showed that bisphosphonates can induce human breast cancer cell death *in vitro* (apoptosis and/or necrosis), which could contribute to their beneficial clinical effects. Thus, bisphosphonates exhibit beneficial effects on bone integrity by reducing bone resorption induced by osteoclasts and maybe also by direct ‘antitumoral’ effects.

In cancer patients treated with bisphosphonates, metastatic breast cancer cells in bone are thus exposed to both bisphosphonates, which can induce their death, and to growth factors, which, in contrast, may stimulate their growth and survival. We investigated the effects of combinations of bisphosphonates and several ‘bone-derived’ growth factors on breast cancer cell survival to examine if bisphosphonates could also inhibit the stimulatory and protective effects of growth factors on breast cancer cells. Our data point to a possibly new mode of action of bisphosphonates in the process of breast cancer-induced osteolysis.

## MATERIALS AND METHODS

### Materials

Media, supplements and plastic culture materials were obtained from Life Technologies SA (Merelbeke, Belgium). Recombinant human insulin-like growth factor types I and II (IGF-I and -II), basic fibroblast growth factor (FGF-2) and epidermal growth factor (EGF) were purchased from R&D Systems (Abingdon, Oxon, UK). Pamidronate (3-amino-1-hydroxypropylidene bisphosphonic acid) and zoledronic acid (2-imidazol-1-yl-1-hydroxyethylidene-1,1 bisphosphonic acid) were provided by Novartis (Basel, Switzerland). Zoledronic acid evidently becomes zoledronate in culture conditions, explaining why we used this last term thoughout the Results. Clodronate (dichloromethylene bisphosphonic acid) and ibandronate (1-hydroxy-3-methylpentylamino-propylidene bisphosphonic acid) were provided by Hoffmann-LaRoche (Basel, Switzerland). MTT (3-[4,5-dimethylthiazol-2-yl]-2,5-diphenyl tetrazolium bromide) reagent and rabbit anti-*β*-actin antibody were obtained from Sigma-Aldrich SA (Bornem, Belgium). Ac-IETD-AMC and Ac-DEVD-AMC were obtained from Pharmingen (Erembodegem-Aalst, Belgium). The 7-amino-4-methylcoumarin was from Bachem Feinchemikalien AG (Bubendorf, Switzerland). The mouse bax and bcl-2 antibodies were purchased from Santa Cruz Biotechnology (Santa Cruz, CA, USA). The phosphospecific antibodies anti-ERK1/2 MAPK, -p38 MAPK and -JNK were purchased from New England Biolabs Inc. (Beverly, MA, USA), and secondary antibodies conjugated with horseradish peroxidase were from Dako (Denmark).

### MTT test

Subconfluent cells were washed twice in PBS before addition of phenol red-free and serum-free medium containing or not the indicated agents (bisphosphonates or growth factors). We previously tested and compared several techniques (MTT test, cell counting, total DNA content and BrdU incorporation) to investigate breast cancer cell survival under serum-free conditions. In this study, we used the MTT test as previously described, and all significant effects were validated by cell counting. We used six replicates for each condition and experiments were repeated at least three times. Results are means±s.e.m. of treated/control ratios.

### Caspase activity

After 3, 8, 15, 24 and 48 h of incubation under serum-free conditions, cells were trypsinised and processed as previously described (Fromigue *et al*, 2000). Briefly, caspases activities were assessed by the cleavage of synthetic fluorogenic substrates containing the amino-acid sequences IETD: Ile-Glu-Thr-Asp (caspase-8), or DEVD: Asp-Glu-Val-Asp (effector caspase -3, -6 and -7) combined to a fluorophore (AMC). MCF-7 cell line is known to be devoid of a caspase-3 activity (Janicke *et al*, 1998), which results in the determination of caspase-6 and -7 activities by the DEVD-AMC substrate. Results, obtained as nmol AMC h^−1^ 10^−6^ cells, are expressed as treated over control ratios.

### Western blotting

Attached cells, collected by trypsinisation, were pooled with the floating cells, lysed in 10 mM Tris-HCl pH 7.4, 20 mM NaCl, 1% NP40, 0.5% deoxycholate Na and 0.1 mM PMSF for 30 min on ice and centrifuged. Proteins were separated on 12% SDS–PAGE and electrotransferred onto PVDF membranes. Filters were blocked in 10 mM Tris-HCl pH 7.4, 150 mM NaCl, 1 mM EDTA, 0.1% Tween-20, 3% BSA and 0.5% gelatin for 3 h, then incubated overnight with primary antibody (1 : 500 for *β*-actin, 1 : 1000 for bax or bcl-2). Filters were washed twice before incubation for 1 h with the secondary antibody conjugated with horseradish peroxidase (1 : 2500). After three final washes, filters were exposed following the enhanced chemiluminescence detection reagent (Amersham, Les Ulis, France) to an autoradiographic film (Kodak, Vilvoorde, Belgium). For detection of the phosphorylation state of p38-, ERK1/2-MAPKs and JNK, subconfluent cells were washed twice in PBS before incubation with phenol red-free and serum-free medium containing bisphosphonates and/or growth factors. After 2, 5, 10 or 30 min, culture media were removed and flasks immersed in dry ice-cold methanol. Cell layers were then lysed as described above. Phospho-specific primary antibodies were used at the dilution 1 : 2000.

### Statistical analysis

Statistical analysis was performed using classical statistical tests (package Super ANOVA; Macintosh, Abacus concepts Inc., Berkeley, CA, USA) with a statistical significance level at least <0.05 (Fisher's PLSD).

## RESULTS

### Dose–response effects of growth factors on breast cancer cells

**Figure 1:**
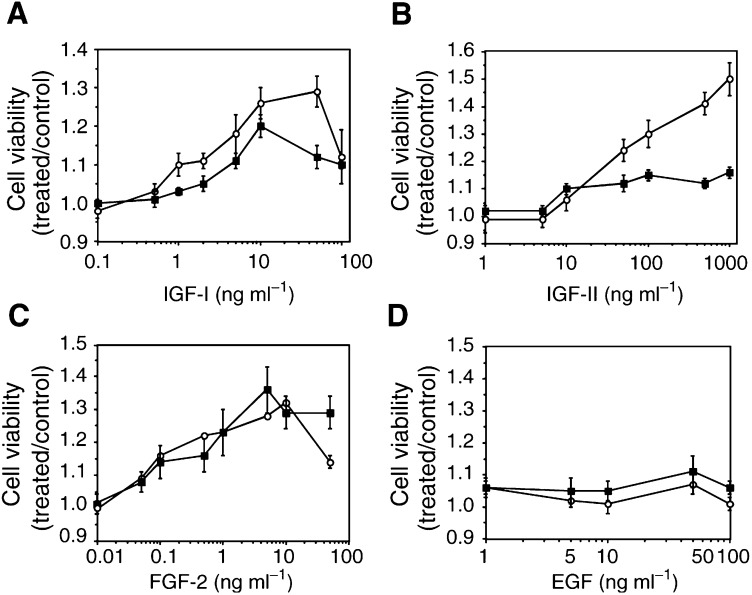
Modulation of breast cancer cell survival by growth factors. Breast cancer cells (○, MCF-7 and ▪, T47D) were incubated for 24 h under serum-free conditions, without or with increasing concentrations of IGF-I, IGF-II, FGF-2 or EGF. Cell viability was evaluated using the MTT test and expressed as treated over control ratios (means±s.e.m., *n*=15–20).

Cell survival was assessed by the MTT test and all significant effects were validated by cell counting (see Materials and methods; data not shown). We checked the effects of four different growth factors on the viability under serum-free conditions of two classical breast cancer cell lines, namely MCF-7 and T47D. Exposure to IGF-I (0.1–100 ng ml^−1^) or IGF-II (1–1000 ng ml^−1^) for 24 h increased cell survival in a dose-dependent manner. IGF-I induced a bell-shaped growth curve with a peak at 10 ng ml^−1^ (up to +26%, *P*<0.005; Figure 1A

). Lower concentrations (1–5 ng ml^−1^) already significantly increased cell viability and concentrations above 50 ng ml^−1^ had lower stimulatory effects. Doses 10–50-fold higher were required for IGF-II to detect stimulatory effects comparable to IGF-I. In contrast to IGF-I, IGF-II did not exhibit a biphasic pattern, but dose-dependently increased MCF-7 cell survival, at least up to 1000 ng ml^−1^ (+50%, *P*<0.05; Figure 1B). IGF-II also dose-dependently improved T47D cell survival and the stimulatory effects persisted for concentrations above 100 ng ml^−1^, then plateaud (+15%, *P*<0.05; Figure 1B). These data confirm that IGFs can increase MCF-7 and T47D cell survival and indicate that IGF-II was about 10-fold less potent than IGF-I in our model. FGF-2 dose-dependently increased MCF-7 and T47D cells survival (up to +36%, *P*<0.05; Figure 1C). Very low concentrations of 0.05 ng ml^−1^ increased cell viability by about 10% (*P*<0.02). The maximal stimulatory effect was observed for concentrations of 5–10 ng ml^−1^ for both cell lines (+30%, *P*<0.001). Higher concentrations did not improve T47D nor MCF-7 cell viability. Lastly, EGF (1–100 ng ml^−1^) did not exert any significant effect on MCF-7 or T47D cell survival (Figure 1D). We thus did not further investigate the possible modulatory effects of bisphosphonates on the effects of this particular growth factor.

### Bisphosphonates inhibit growth factors' proliferative effects

**Figure 2:**
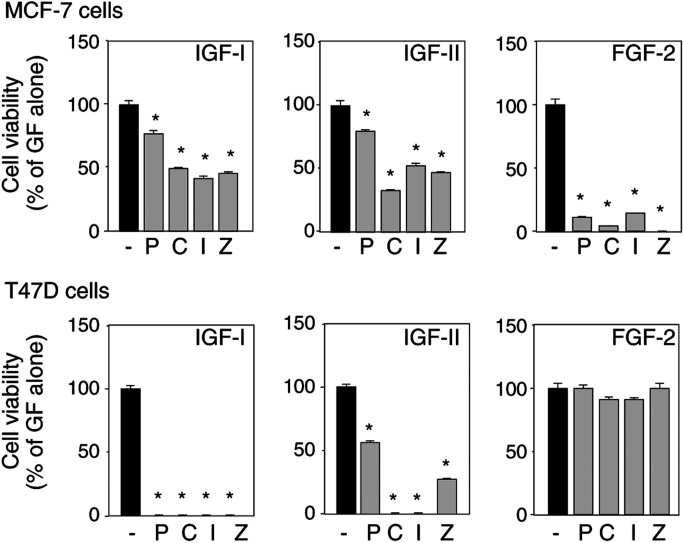
Bisphosphonates reduce growth factors' stimulatory effects on human breast cancer cell survival. MCF-7 cells (upper panel) and T47D cells (lower panel) were incubated for 24 h under serum-free conditions, in the presence of 10^−6^ M pamidronate (P), clodronate (C), ibandronate (I) or zoledronate (Z), and of IGF-I (10 ng ml^−1^), IGF-II (100 ng ml^−1^) or FGF-2 (5 ng ml^−1^). Cell viability was evaluated by the MTT test and expressed as the percentage of growth factors' effects alone (means±s.e.m., *n*=18–20). ^*^:*P*<0.05 *vs* growth factor alone.

We then investigated the effects of bisphosphonates in combination with growth factors on breast cancer cell viability under serum-free conditions. Bisphosphonates were all tested at the concentration of 10^−6^ M and inhibited cell viability by up to 27% in MCF-7 cells and by up to 36% in T47D cells (see Fromigué *et al*, 2000). IGF-I, IGF-II and FGF-2 were used at concentrations exhibiting optimal or suboptimal stimulatory effects, that is, 10 ng ml^−1^ for IGF-I, 100 ng ml^−1^ for IGF-II and 5 ng ml^−1^ for FGF-2 (see Figure 1). In MCF-7 cells, the most impressive effects of bisphosphonates were observed with FGF-2 (Figure 2

, upper panel) since all four bisphosphonates almost completely abolished the FGF-2 stimulatory effects (86–99% inhibition). The effects of IGFs were less inhibited by bisphosphonates (reduction of 20–68% of their proliferative effect). For the T47D cell line, we found a quite different pattern since FGF-2-treated cells were not affected by bisphosphonates (Figure 2, lower panel), whereas IGF-I effects were completely abolished by all four tested bisphosphonates. Clodronate and ibandronate also fully inhibited IGF-II stimulatory effects on T47D cell survival, whereas pamidronate and zoledronate partly reduced (by 43 and 72%, respectively) IGF-II effects (Figure 2, lower panel). These data show that bisphosphonates can completely or partly inhibit growth factors' stimulatory effects on breast cancer cell survival. No marked differences could be noticed between the four bisphosphonates except that pamidronate was often the least potent compound.

### Differential effects of bisphosphonates and growth factors on apoptosis

**Figure 3:**
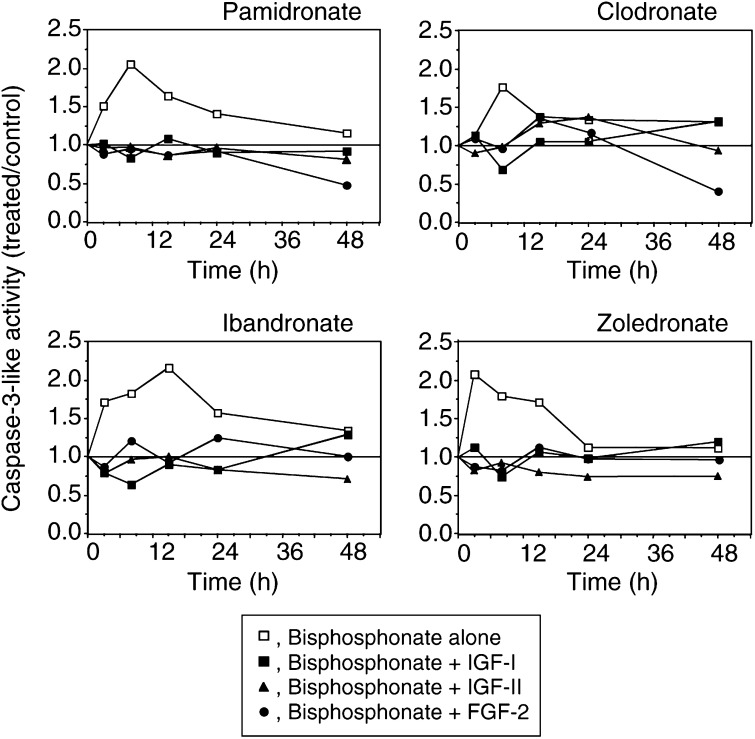
Modulations of effector caspases activity in MCF-7 cells. Subconfluent MCF-7 cells were incubated in the presence of 10^−6^ M bisphosphonates and IGF-I (10 ng ml^−1^), IGF-II (100 ng ml^−1^) or FGF-2 (5 ng ml^−1^) for up to 48 h. Effector caspases (caspase -6, -7) activity was determined at different time points using a synthetic fluorogenic substrate (DEVD-AMC, see Materials and methods). Results are shown as treated over control ratios (*n*=3).

We previously reported that bisphosphonates reduce breast cancer cell survival by inducing both programmed cell death (apoptosis) and direct necrosis in the MCF-7 cell line, whereas necrosis is the main mechanism involved in the reduction of T47D cell survival (Fromigue *et al*, 2000). The bisphosphonate-induced apoptotic process in MCF-7 cells is characterised by a time-dependent activation of effector caspases. We investigated the modulation of the activity of initiator (caspase-8) and effector caspases (caspase-6 and -7) in MCF-7 cells incubated in the presence of bisphosphonates and growth factors. No modulation of caspase-8 activity was detected in bisphosphonates and/or growth factors treated cells (data not shown). We found that neither IGF-I nor IGF-II nor FGF-2 significantly modified the basal levels of effector caspases activity in MCF-7 cells at any time (from 3 to 48 h; data not shown). However, when a bisphosphonate and a growth factor were simultaneously added in MCF-7 cells culture medium, we found a marked reduction in bisphosphonate-induced stimulation of effector caspases activity (Figure 3

). These data suggest that growth factors could attenuate bisphosphonate-induced MCF-7 cells apoptosis by lowering the caspases induction.

**Figure 4:**
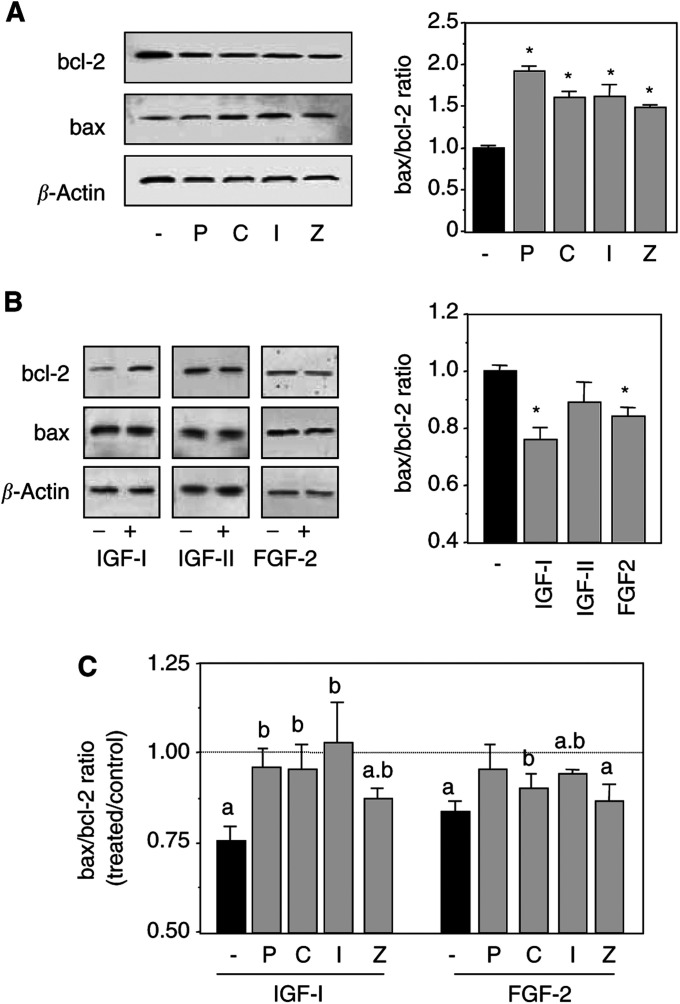
Modulations of the bax/bcl-2 ratio in MCF-7 cells. Subconfluent MCF-7 cells were incubated for 24 h, under serum-free conditions, in the absence or presence of 10^−6^ M pamidronate (P), clodronate (C), ibandronate (I), zoledronate (Z), 10 ng ml^−1^ IGF-I or 5 ng ml^−1^ FGF-2. Bax and bcl-2 expression levels were determined by Western blot and corrected for *β*-actin levels. Results are expressed as means±s.e.m. of the bax/bcl-2 ratio (*n*=4–10). ^*^:*P*<0.05 *vs* control. (**A**) Blots and derived ratios under bisphosphonates treatment. (**B**) Blots and derived ratios under growth factors treatments. (**C**) Relative intensity of signals under combinations of growth factors (IGF-I or FGF-2) and bisphosphonates. a: *P*<0.05 *vs* control; b: *P*<0.05 *vs* growth factor alone.

Other intracellular effectors are involved in the induction of apoptosis. The ratio between proapoptotic proteins (such as bax) and antiapoptotic proteins (such as bcl-2) determines whether a cell will undergo apoptosis or will be protected from it (Adams and Cory, 1998). We investigated the effects of growth factors and of bisphosphonates, alone or in combination, on bax and bcl-2 protein levels in MCF-7 cells, and found that all four bisphosphonates increased the bax/bcl-2 ratio (Figure 4A

) whereas IGF-I and FGF-2 significantly decreased the same ratio (Figure 4B). The effects of IGF-II were not significant. When bisphosphonates were combined with IGF-I or FGF-2, we observed a reduction in the antiapoptotic effect of growth factors on MCF-7 cells (Figure 4C). IGF-I protective effects were reduced by 50–100% (*P*<0.05) by bisphosphonates and the ones of FGF-2 by 19–75% (*P*<0.05). These data indicate that bisphosphonates could reduce the protective effects of growth factors on MCF-7 cell survival by significantly reducing their antiapoptotic potential.

### Bisphosphonates and FGF-2 modulate cell survival signalling pathways

**Figure 5:**
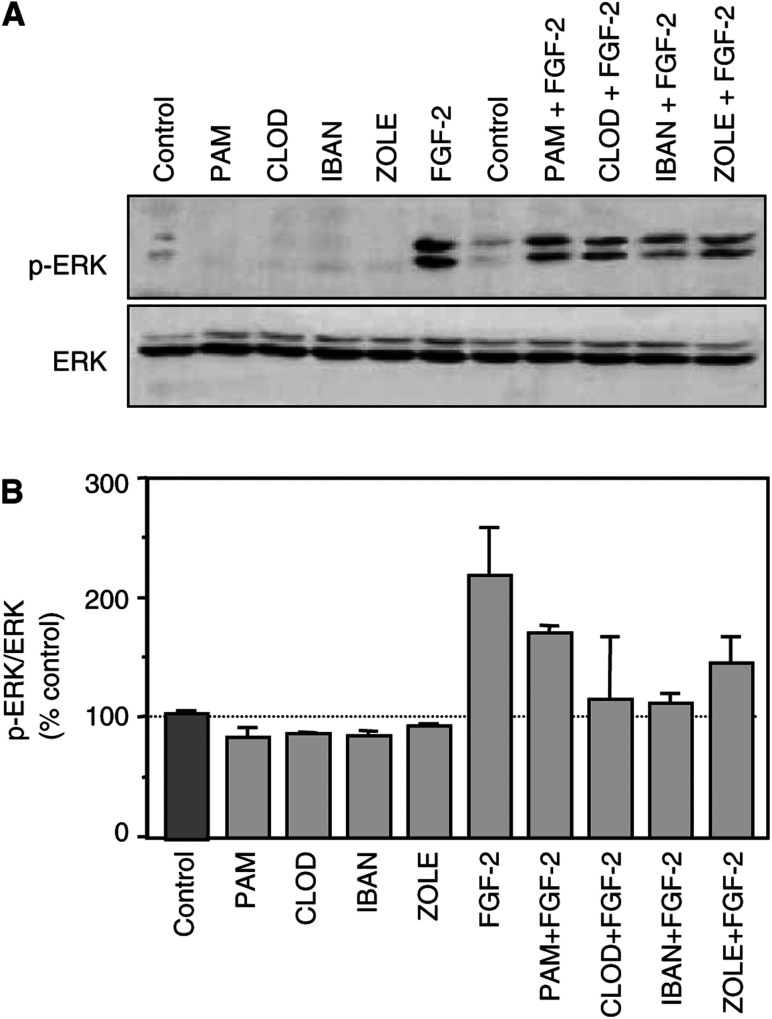
Modulation of the ERK-MAPK phosphorylation status in MCF-7 cells. (**A**) MCF-7 cells were incubated for 10 min with bisphosphonates (10^−6^ M) and/or FGF-2 (5 ng ml^−1^) in phenol red-free and serum-free medium. Cell lysates were analysed by Western blot for phospho-p42/p44 and total p42 expression as described in Materials and methods section. (**B**) Relative intensity of signals, after scan and software analyses (ImageQuant). Results are expressed as percentages of controls. PAM=pamidronate; CLOD=clodronate; IBAN=ibandronate and ZOLE=zoledronate.

Cell survival is classically upregulated by growth factors such as IGFs or FGF-2 via intracellular signalling pathways involving mitogen-activated protein kinases (MAPK) or stress-activated protein kinases (SAPK). We investigated by Western blotting the effects of bisphosphonates and/or growth factors on phosphorylation states of these kinases in MCF-7 breast cancer cells. No modulatory effects on p38-MAPK or JNK phosphorylation levels could be detected under bisphosphonates or/and growth factors treatments (data not shown). By contrast, IGFs weakly stimulated ERK1/2-MAPK phosphorylation (by about 10% after 5–10 min, data not shown), whereas FGF-2 induced a marked increase in ERK1/2-MAPK phosphorylation status (by a maximum of 2.3-fold compared to untreated cells after 10 min; Figure 5

). In contrast, all four bisphosphonates induced a slight decrease in phospho-ERK1/2 rates (by 7–16%; Figure 5). When FGF-2 and bisphosphonates were combined, the effects of FGF-2 were markedly or completely inhibited (*P*<0.05). These initial data suggest that bisphosphonates and FGF-2 can modulate in opposite directions the phosphorylation status of common signalling pathways such as MAPK, which is involved in the balance between induction of and protection against cell death.

## DISCUSSION

The high concentrations of growth factors in bone matrix provide a fertile ‘soil’ for metastatic breast cancer cells (hypothesis proposed by Paget, 1889). When tumour cells arrive in bone, a vicious cycle arises since they stimulate osteoclast-induced bone resorption, enhancing the supply of bone-derived growth factors in the bone microenvironment. This may explain why bone tissue is the most frequent organ in which breast cancers develop distant metastases (Boyce *et al*, 1999; Yoneda *et al*, 2000). IGFs are the most abundant growth factors stored in bone matrix (Mohan and Baylink, 1996) and, more importantly, are potent survival factors for a wide range of tumour cells, notably breast cancer cells (Dunn *et al*, 1997), by exerting both mitogenic and antiapoptotic effects (Osborne *et al*, 1990; Baserga *et al*, 1994). The role of IGF-I as a survival factor for cells in culture was first demonstrated in IL-3-dependent haemopoietic cells (Rodriguez-Tarduchy *et al*, 1992). Removal of IL-3 results in cell apoptosis, which can be blocked by adding IGF-I to cell cultures. In the same way, FGF-2 can stimulate cancer cell proliferation and prevent cell death (Miyake *et al*, 1997). There is thus substantial evidence to indicate that IGFs and FGF-2 are critical survival factors for cancer cells, and that metastases development is indeed dependent on such survival signals. We first examined the modulation of breast cancer cell survival in the presence of IGF-I, IGF-II, FGF-2 or EGF, and we confirmed that, except for EGF, these growth factors promoted MCF-7 and T47D cell survival *in vitro* under serum-free conditions. In agreement with other authors' data (McCarthy *et al*, 1989), we observed that IGF-II was about 10-fold less potent than IGF-I to increase breast cancer cell survival under serum-free conditions.

Bisphosphonates are synthetic compounds that preferentially accumulate in bone (Sato *et al*, 1991) by their strong affinity for calcified matrix. They are powerful inhibitors of osteoclast-mediated bone resorption (Fleisch, 1991, 1997a; Russell and Rogers, 1999), and they significantly reduce skeletal morbidity in advanced breast cancer patients (Body, 2000). Bisphosphonates can also decrease the number of bone metastases (Hall and Stoica, 1994; Powles *et al*, 2002). Moreover, we and others recently demonstrated that, *in vitro*, bisphosphonates irreversibly reduce breast cancer cell viability by inducing cell death, either by apoptosis or by direct cell necrosis (Fromigue *et al*, 2000; Senaratne *et al*, 2000; Jagdev *et al*, 2001a). The exact mechanism of action of bisphosphonates is unknown. We checked the hypothesis of a cation chelation by testing increasing concentrations of EDTA on MCF-7 cell viability and apoptosis. No significant effect was detected even at the highest concentration (10^−4^ M; data not shown). Recent data go along the same line (Jagdev *et al*, 2001b) and led us to discard the possibility that the observed effects are due to simple cation chelation by the bisphosphonates.

It is difficult to know or to evaluate the concentrations of bisphosphonates that are reached in the bone microenvironment *in vivo*. Studies in rats revealed that concentrations as high as 10^−4^ M or 10^−3^ M can be reached in resorption lacunae (Sato *et al*, 1991). The concentrations we chose (10^−6^ M) could thus be easily reached in the skeleton, and it did not induce strong toxicity *in vitro* as it can be observed for concentrations of 10^−4^ or 10^−3^ M (Fromigue *et al*, 2000). We showed here that this relatively low concentration was able to block growth factors' effects, and to inhibit the ‘protective’ effect of growth factors on breast cancer cell survival. The effects of bisphosphonates that we observed were thus obtained with relatively low concentrations of bisphosphonates, in contrast to earlier works that reported the requirement for higher concentrations, between 20 and 1000 *μ*M, to exhibit antitumour effects (Senaratne *et al*, 2000; Lee *et al*, 2001; Jagdev *et al*, 2001b).

Our hypothesis is thus that in bisphosphonate-treated patients, metastatic breast cancer cells in bone are influenced by various growth factors, which may promote cell survival and/or cell growth, but they can also be in contact with bisphosphonates that might induce their death. A ‘competition’ between these two opposite effects could thus modulate breast cancer cell proliferation and death. Very few data are available on the potential interactions between bisphosphonates and growth factors, but it was recently reported that administration of bisphosphonates to patients with bone metastases significantly decreases serum FGF-2 concentrations (Zimering, 2002). It has also been shown that pamidronate inhibits the effects of GH and decreases IGF-I levels in rats (Kapitola *et al*, 2000), or inhibits the effects of G-CSF on bone cells (Suzuki *et al*, 1999). Our first experiments showed that when both growth factors and bisphosphonates were simultaneously added to cell cultures, the stimulatory effects of growth factors on cell survival were markedly decreased (for IGFs) or even abolished (for FGF-2). The increased cell survival observed in IGFs-treated cells can be explained by an inhibition of apoptosis, confirmed for example by a decrease in the bax/bcl-2 ratio (Jung *et al*, 1996; Dunn *et al*, 1997; Xu *et al*, 1997). Bisphosphonates could counterbalance these protective effects of bone-derived growth factors on human breast cancer cell survival. On the other hand, we confirmed a time-dependent increase in caspases activity under bisphosphonate treatment, as previously described (Fromigue *et al*, 2000). This stimulation was almost completely abolished when MCF-7 cells were incubated with both growth factors and bisphosphonates.

In *in vivo* conditions, due to bisphosphonate-induced decrease in bone turnover, the release of bone-derived growth factors should be diminished, resulting in a less favourable microenvironment for cancer cells growth. This would come in addition to the inhibition by bisphosphonates of the protective effects of growth factors on cancer cells that we report here. Bisphosphonates and growth factors thus appear to be concurrent compounds for tumour cell survival in bone tissue. There might be a simple balance between opposite effects or a real antagonism between these two classes of compounds. We thus tried in the last part of this report to start to characterise such possible interactions. The exact intracellular mechanisms of action of bisphosphonates are still to be further delineated and our preliminary data could point to a new molecular mode of action of bisphosphonates.

In this study, we tested four structurally different bisphosphonates, known to exhibit variable potencies in bone resorption inhibition. Indeed, clodronate and pamidronate are bisphosphonates of first/second generations. In contrast, ibandronate and zoledronic acid are last generation bisphosphonates that represent the two most potent compounds (Hiraga *et al*, 2001; Jagdev *et al*, 2001b; Rosen *et al*, 2001; Body, 2003; Lipton, 2003). Despite their marked differences in potency to inhibit bone resorption *in vitro* or *in vivo*, we did not observe major differences between the potency of these four compounds in our *in vitro* experiments. It is now accepted that nitrogen-containing bisphosphonates can inhibit osteoclast activity through the mevalonate pathway (Luckman *et al*, 1998; Benford *et al*, 1999; van Beek *et al*, 1999; Coxon *et al*, 2000). In our model, it appears likely that modulations of the mevalonate pathway by nitrogen-containing bisphosphonates cannot entirely explain the effects we observed on human breast cancer cells since all four tested compounds reduced bone growth factors' effects on cell survival at about the same degree. Indeed, pamidronate, ibandronate and zoledronate are nitrogen-containing bisphosphonates, which interfere with the mevalonate pathway, whereas clodronate does not and is actually metabolised into a toxic ATP analogue (Frith *et al*, 1997). A common characteristic to these four structurally different compounds is evidently the presence of two phosphate groups, which could suggest that bisphosphonates could interact with intracellular phosphorylation signalling pathways (including kinases, phosphorylation reactions or phosphatase activities). Along that line, some authors already reported modulations of protein–tyrosine phosphatase activity by alendronate in osteoclasts (Schmidt *et al*, 1996) and of the MAPK pathway in osteoblasts by six different bisphosphonates (Plotkin *et al*, 1999). It is also well known that growth factors modulate cell metabolism through interactions with specific kinase receptors and intracellular signal transduction through MAPK or PI-3K pathways (Dufourny *et al*, 1997; Imai and Clemmons, 1999). We evaluated the phosphorylation state of JNK, ERK1/2 and p38 MAPK after incubation with bisphosphonates and/or growth factors. No modification in JNK and p38 phosphorylation levels was detectable by Western blot, but the ERK1/2 pathway was clearly affected, suggesting a modulation of cell survival and not a response to stress (Xia *et al*, 1995). As expected, FGF-2 induced a marked increase in ERK1/2 phosphorylation status but, interestingly, bisphosphonates decreased ERK1/2 phosphorylation and attenuated FGF-2 effects. Further experiments should evidently be performed, but these preliminary data suggest another possible mechanism of intracellular action of bisphosphonates.

In conclusion, in addition to the previously demonstrated direct ‘antitumour’ effects of bisphosphonates, the present report indicates that bisphosphonates might antagonise the stimulatory effects of growth factors on the proliferation of breast cancer cells and counterbalance their protective effects on breast cancer cell death. Changes in intracellular phosphorylation transduction pathways could partly explain our observations. Maybe importantly from a clinical point of view, these effects could contribute to the beneficial activity of bisphosphonates and appear to indicate that the mechanism of inhibition of tumour-induced osteolysis by bisphosphonates is much more complex than a ‘simple’ antiosteoclast activity. Our findings may thus represent a novel mechanism of action of bisphosphonates in the process of inhibition of tumour-induced osteolysis.
